# Inflammatory ultrasound features as prognostic factors of pain and functional outcomes following intra‐articular platelet‐rich plasma in knee osteoarthritis

**DOI:** 10.1111/1756-185X.14781

**Published:** 2023-06-13

**Authors:** Win Min Oo, James Linklater, Kim L. Bennell, Shirley P. Yu, Vicky Duong, David J. Hunter

**Affiliations:** ^1^ Rheumatology Department, Royal North Shore Hospital and Sydney Musculoskeletal Health, Kolling Institute University of Sydney Sydney New South Wales Australia; ^2^ Department of Physical Medicine and Rehabilitation, Mandalay General Hospital University of Medicine, Mandalay Mandalay Myanmar; ^3^ Department of Musculoskeletal Imaging Castlereagh Sports Imaging Centre St. Leonards, Sydney New South Wales Australia; ^4^ Department of Physiotherapy, Centre for Health, Exercise and Sports Medicine, School of Health Sciences, Faculty of Medicine Dentistry & Health Sciences The University of Melbourne Melbourne Victoria Australia

**Keywords:** disease‐modifying osteoarthritis drugs, inflammation, osteoarthritis, platelet‐rich plasma, prognosis, ultrasonography, ultrasound

## Abstract

**Aim:**

To explore inflammatory ultrasound predictors of improvements in pain and function over 2, 6, and 12 months following administration of intra‐articular platelet‐rich plasma (PRP) in knee osteoarthritis (OA).

**Method:**

Patients with painful mild–moderate radiographic knee OA from a subset of the RESTORE RCT underwent ultrasound assessment according to the standardized OMERACT scanning protocol to detect inflammatory features such as synovitis, synovial hypertrophy, and effusion with power Doppler. The study knee was treated with 3 once‐weekly PRP injections obtained after centrifugation at 1500 *g* for 5 min. Numerical Rating Score (NRS), Intermittent and Constant Osteoarthritis Pain (ICOAP) questionnaire, and the Western Ontario and McMaster Universities Arthritis Index (WOMAC) function sub‐score were used to measure pain and functional severity. Separate linear regression models were performed to determine whether baseline ultrasound‐detected features of inflammation predicted the improvement in pain and function following PRP injection in both unadjusted and adjusted models for confounders.

**Results:**

Forty‐four participants were included, with 25 (56.8%) being female. In an unadjusted model, higher OMERACT scores for inflammatory features such as global synovitis and/or effusion were significantly associated with greater improvement in all outcomes measured at 2 months but not at 6 and 12 months for pain measures. Only global synovitis showed significant association with functional improvement at 2 and 12 months. Similar findings were observed in the adjusted model.

**Conclusion:**

Ultrasound indices of knee inflammation predicted short‐term improvements in pain severity and both short‐ and longer‐term improvements in function following intra‐articular PRP injection.

**Plain Language Summary**

1. Inflammatory ultrasound features may have potential prognostic value in predicting improvement in pain and function outcomes after intra‐articular platelet‐rich plasma administration in knee osteoarthritis.

## 1 INTRODUCTION

Osteoarthritis (OA) is the most prevalent joint disease^1^ and was ranked as the 15th highest contributor to global disability in 2019.^2^ The pooled global prevalence of knee OA in individuals aged 40 years or older is 23%,^3^ with the number rising globally by 48% from 1990 to 2019.^2^ The healthcare costs of knee OA are predicted to double by 2040.^4^ Currently available drug therapy is limited to improvement in pain and function^5^ with no regulatory‐approved disease‐modifying osteoarthritis drugs.^6^ It is important to note that OA is an incredibly heterogeneous disease and current OA treatment options are largely symptomatic; therefore, an insight into what pathologies of OA can predict the symptomatic improvement following certain interventions is an important research and therapeutic goal.

One treatment option that can potentially address the symptomatic, as well as underlying, OA pathobiological processes, is platelet‐rich plasma (PRP), obtained from autologous blood.^7^, ^8^ In preclinical animal studies, the release of a large number of mediators by activation of platelets in PRP (granule secretion) has mainly a homeostatic effect with anabolic and anti‐inflammatory effects on joint tissues.^9^, ^10^, ^11^ The recent meta‐analyses reported favorable symptomatic benefits following intra‐articular PRP administration in knee OA.^8^, ^12^, ^13^ The French National Authority for Health recommendations recently issued a consensus statement that intra‐articular injections of PRP may be proposed for early to moderate knee OA after the failure of well‐conducted oral and topical symptomatic treatments and appropriate physiotherapy.^14^ However, it was noted in their recommendations that few studies had examined predictive factors associated with outcomes following PRP injections.

Ultrasound is becoming popular because of its wide availability, relatively low cost, visibility of soft‐tissue structures of the joints, and safety.^15^ Due to these advantages, an outcome measure in rheumatology (OMERACT) ultrasound score for knee OA was developed through international consensus in 2016^16^ and showed moderate to excellent reliability^16^, ^17^ and significant associations with pain, functional impairment and imaging findings.^17^ Although ultrasound has the capacity prognostically to ascertain people who might have more inflammatory features and therefore might be more amenable to PRP therapy,^15^ there is currently no literature examining it as a predictor for pain and functional outcomes.^18^ Therefore, the aim of this study is to explore potential inflammatory predictors detected by ultrasound for improvement in pain and function over 2, 6, and 12 months following intra‐articular PRP injection in knee OA.

## 2 MATERIALS AND METHODS

### 2.1 Study design and participant selection

Pain and function data were obtained from a sub‐sample of the PRP group at the Sydney site of the Australian RESTORE trial (platelet‐Rich plasma as a symptom‐ and disEaSe‐modifying Treatment fOR knee osteoarthritis; trial registration no: ACTRN12617000853347). The RESTORE trial was a two‐arm, parallel‐group, participant‐, care provider‐, outcome assessor‐blind, placebo (saline)‐controlled randomized clinical trial examining the effect of three once‐weekly intra‐articular PRP administrations over 12 months in patients with knee OA.^19^ Detailed information about the selection criteria and methodology have been published in the study protocol.^20^ This PRP cohort included a convenience subsample that underwent baseline ultrasound scans between September 2017 and February 2019. All participants provided written informed consent. This study was approved by the Northern Sydney Local Health District Human Research Ethics Committee (HREC/16/HAWKE/430).

### 2.2 PRP preparation

A commercially available product (Regen Lab SA, Le‐Mont‐sur‐Lausanne, Switzerland), which yields a platelet concentration factor of 1.6–5 times over whole blood values, was used to obtain PRP.^21^ The blood collection tube was gently inverted several times to mix the anti‐coagulant with the blood sample collected. Then the mixture was centrifuged at 1500 *g* for 5 min by the study nurse. After centrifugation, the tubes were gently agitated to ensure all visible platelet aggregates were detached from the separating gel and the tube wall, and 5 mL was gently withdrawn into a 10‐mL Luer‐lock syringe attached to a 23‐gauge (38 mm) needle. A video of the PRP preparation procedure can be found at https://www.youtube.com/watch?v=Cq8UlbL18Yg&feature=youtu.be

### 2.3 PRP administration

Five milliliters of PRP was injected into the knee joint under ultrasound guidance using a medial patellofemoral approach. Following the injection, passive knee flexion and extension was performed five times, with the participant observed resting for 10 min thereafter. Participants were administered with three intra‐articular knee injections performed at weekly intervals, in line with commonly used injection protocols for knee OA.^22^, ^23^

### 2.4 Ultrasound evaluation

At baseline, a physician operator (WMO, 6 years of musculoskeletal ultrasound experience and certified with musculoskeletal ultrasound in rheumatology [RhMSUS] by the American College of Rheumatology) blinded to clinical outcomes, performed the ultrasound scans of the study knee according to the standardized EULAR scanning methods^24^ and used the OMERACT knee ultrasound OA atlas^16^ for grading the severity of inflammation. A multi‐frequency linear 14L5 transducer (using 10 MHz) of Aplio Platinum 500 machine (Toshiba, Tokyo, Japan), was used to detect inflammatory manifestations of knee OA visualized on ultrasound: semi‐quantitative scores for (a) synovitis (0–3) (combined synovial hypertrophy and effusion), binary scores (0–1) for (b) synovial hypertrophy ≥4 mm, (c) effusion ≥4 mm, and (d) power Doppler signals in the suprapatellar recess in a longitudinal plane, medial and lateral parapatellar recesses in a transverse plane respectively.^17^ In each participant, the sum of the synovitis scores in the suprapatellar and parapatellar recesses (range 0–9), was calculated as an indicator of global inflammatory changes at a single knee joint level.^25^ The intra‐rater and inter‐rater reliabilities were good to excellent and are reported elsewhere.^17^ The application specialist from Toshiba company optimized the machine setting, with the parameters of gray‐scale gain = 85%, probe frequency = 10 MHz, Doppler frequency = 6Mhz, Doppler gain = 40%, pulse repetition frequency = 14.8 kHz, and wall filter = 5. The ultrasound operator was not allowed to change these, except for depth and focus, throughout the study.

The maximum score approach of each single ultrasound pathology (ie using the highest score of the same ultrasound features such as synovitis from different scanned sites if two or more sites were scanned)^26^ was applied to examine the ultrasound predictors for the response to PRP injection.

### 2.5 Clinical assessment of pain and function

Average overall knee pain severity over the last week was measured at baseline, 2, 6, and 12 months, using an 11‐point numerical rating score (NRS) with terminal descriptors ‘no pain’ (score 0) and ‘worst pain possible’ (score 10), with the highest scores denoting the worst pain.^27^ The test–retest reliability and validity were confirmed in a recent study in knee OA (intraclass correlation coefficient = .95 (.93–.96) and Pearson's correlation coefficient = .94).^28^ The Intermittent and Constant Osteoarthritis Pain (ICOAP) questionnaire was administered at baseline, and 2 and 12 months. It includes five items assessing constant knee pain in the previous week, and six items assessing intermittent knee pain in the previous week (higher score denotes severe pain).^29^ The ICOAP demonstrates excellent test–retest reliability as well as validity with similar instruments used to measure OA pain.^30^ The Western Ontario and McMaster Universities Arthritis Index (WOMAC) function sub‐score was obtained from 17 questions regarding knee function in the last week (higher scores indicate worse functional limitations)^31^ and was evaluated at baseline, and 2 and 12 months.

### 2.6 Statistics

Descriptive statistics of categorical or continuous variables were expressed as frequencies, means and standard deviations (SD) as appropriate. The OMERACT ultrasound scoring system consists of four separate ultrasound scores indicative of inflammation. Therefore, the associations were examined separately for these ultrasound scores. Separate linear regression models were fitted with each ultrasound feature as a predictor of PRP response in terms of improvement in pain or function (from baseline to each time point of follow ups) in participants who received PRP, in both unadjusted and adjusted models. In the adjusted model, adjustment was made for age, gender, body mass index, duration of disease, radiographic Kellgren–Lawrence Grade (KLG), and respective baseline outcomes as covariates. All statistics were conducted with SPSS version 23 (IBM, Armonk, NY, USA) and a significant association/correlation was defined as a *p* value less than .05.

## 3 RESULTS

### 3.1 Demographic, clinical characteristics, ultrasound findings

Forty‐four patients out of 72 in the PRP arm in Sydney were included in the analysis. Mean age±SD of the cohort was 61.1 ± 6.4 years, with 25 (56.8%) being female; body mass index was 27.5 ± 5.5 kg/m^2^, pain was 5.7 ± 1.5 on an NRS scale, 6.4 ± 3.7 on the ICOAP Constant pain score, 10.3 ± 4.0 on the ICOAP Intermittent pain score, and 26.9 ± 11.7 on the WOMAC function sub‐score. These baseline characteristics were similar to the overall study population of the RESTORE study.^19^ More than half of participants had KLG III, and 97.7%, 63.6%, 47.7%, and 43.2% showed ultrasound synovitis grade 1 or above, presence of effusion, synovial hypertrophy, and power Doppler signals, respectively (Table 1).

**TABLE 1:** Baseline clinical, radiographic, and ultrasound data of study participants.

	Number (%)	Mean (SD)/Median (interquartile range)
Population	44	
Age, years		61.1 ± 6.4
Female	25 (56.8)	
Body mass index		27.5 ± 5.5
Disease duration, years		7.6 ± 8.4
NRS Average pain		5.7 ± 1.5
ICOAP Continuous		6.4 ± 3.7
ICOAP Intermittent		10.3 ± 4.0
WOMAC Function subscore		26.9 ± 11.7
Radiological scores		
Kellgren–Lawrence grade		3 (2–3)
II	17 (38.6)	
III	27 (61.4)	
Ultrasound OMERACT Scores		
Synovitis grade		2 (0–3)
0	1 (2.3)	
I	9 (20.5)	
II	16 (36.4)	
III	18 (40.9)	
Global synovitis		6 (0–9)
Effusion (+)	28 (63.6)	
Synovial hypertrophy (+)	21 (47.7)	
PD (+)	19 (43.2)	

Abbreviations: NRS, Numerical Rating Scale; OMERACT, Outcome Measure in Rheumatology; PD, power Doppler; WOMAC, Western Ontario and McMaster Universities Osteoarthritis.

### 3.2 Ultrasound predictors for improvement in pain

There was an improvement in NRS average pain score in the PRP cohort over 2 months (−1.7 ± 1.8), 6 months (−1.6 ± 2.6) and 12 months (−1.4 ± 2.4) (Table 2). On the unadjusted model, OMERACT scores of global synovitis and effusion were significantly associated with improvement in pain severity at 2 months (Table 3), ie, a pain improvement of 1.11 units (β coefficient 1.11, 95% CI 0.08–2.15) was found when effusion was present (0–1). The unadjusted results also showed a significant association of global synovitis with pain improvement at 6 months. However, all these significant results disappeared on adjusted analysis. No ultrasound pathologies showed significant association with this outcome in 12‐month follow up in either unadjusted or adjusted models.

**TABLE 2:** Changes of outcome scores over 2, 6 and 12 months.

Outcome scores	Baseline value	Change in 2 months	Change in 6 months	Change in 12 months
NRS Average pain (0–10)	5.7 ± 1.5	−1.7 ± 1.8	−1.6 ± 2.6	−1.4 ± 2.4
ICOAP Continuous (0–20)	6.4 ± 3.7	−1.8 ± 4.4	–	−1.81 ± 4.5
ICOAP Intermittent (0–24)	10.3 ± 4.0	−1.6 ± 5.6	–	−1.6 ± 6.1
WOMAC Function (0–68)	26.9 ± 11.7	−4.2 ± 10.9	–	−5.6 ± 13.0

Abbreviations: ICOAP, Intermittent and Constant Osteoarthritis Pain score; NRS, Numerical Rating Scale; SD, standard deviation; WOMAC, Western Ontario and McMaster Universities Osteoarthritis.

**TABLE 3:** Association of OMERACT ultrasound knee osteoarthritis scores with change in numerical rating scale average knee pain.

Ultrasound pathologies	Grading score	Unadjusted β (95% CI)			Adjusted β (95% CI)^a^		
		2 months	6 months	12 months	2 months	6 months	12 months
Synovitis	0–3	0.58 (−0.07, 1.22)	0.66 (−0.31, 1.63)	0.74 (−0.0.15, 1.64)	0.55 (−0.19, 1.29)	0.70 (−0.34, 1.74)	0.97 (−0.09, 2.03)
Global synovitis	0–9	**0.24 (0.03, 0.46)**	**0.31 (0.01, 0.63)**	0.22 (−0.10, 0.53)	0.26 (−0.01, 0.53)	0.32 (−0.09, 0.72)	0.30 (−0.11, 0.70)
Synovial hypertrophy	0–1	0.64 (−0.45, 1.73)	0.35 (−1.29, 1.99)	−0.71 (−2.24, 0.82)	0.72 (−0.65, 2.09)	0.56 (−1.33, 2.45)	−0.67 (−2.68, 1.34)
Effusion	0–1	**1.11 (0.08, 2.15)**	1.25 (−0.40, 2.90)	−0.09 (−1.67, 1.50)	1.20 (−0.08, 2.47)	1.22 (−0.62, 3.06)	−0.10 (−2.12, 1.86)
Power Doppler	0–1	0.00 (−1.12, 1.12)	0.10 (−1.55, 1.75)	0.21 (−1.34, 1.75)	0.01 (−0.93, 0.95)	−0.13 (−1.50, 1.21)	0.10 (−1.92, 2.13)

*Note:* Bold values for significant test, *p* < .05.

^a^ Adjusted for adjusting for age, gender, body mass index, duration of disease, radiographic Kellgren–Lawrence grade, and baseline symptomatic outcomes as covariates.

An improvement in ICOAP Constant pain score was revealed over 2 months (−1.8 ± 4.4) and 12 months (−1.81 ± 4.5) (Table 2). Both unadjusted and adjusted analyses showed a significant association of all ultrasound pathologies except for power Doppler at 2‐month follow up (Table 4). However, no significant associations existed in 12‐month follow up.

**TABLE 4:** Association of OMERACT ultrasound knee osteoarthritis scores with change in Intermittent and Constant Osteoarthritis Pain score Constant pain.

Ultrasound pathologies	Grading score	Unadjusted β (95% CI)		Adjusted β (95% CI)^a^	
		2 months	12 months	2 months	12 months
Synovitis	0–3	**1.78 (0.30, 3.26)**	0.97 (−0.66, 2.60)	**2.26 (0.57, 3.96)**	1.35 (−0.44, 3.14)
Global synovitis	0–9	**0.79 (0.28, 1.29)**	0.44 (−0.13, 1.01)	**1.07 (0.44, 1.69)**	0.56 (−0.12, 1.24)
Synovial hypertrophy	0–1	**2.51 (0.03, 5.00)**	0.38 (−2.35, 3.11)	**3.75 (0.58, 6.93)**	0.96 (−2.17, 4.09)
Effusion	0–1	**3.56 (1.08, 6.03)**	1.77 (−1.01, 4.56)	**4.67 (1.63, 7.71)**	2.62 (−0.64, 5.88)
Power Doppler	0–1	0.98 (−1.63, 3.60)	0.87 (−1.88, 3.61)	1.69 (−0.37, 3.75)	1.87 (−0.60, 3.80)

*Note:* Bold values for significant test, *p* < .05.

^a^ Adjusted for adjusting for age, gender, body mass index, duration of disease, radiographic Kellgren–Lawrence Grade, and baseline symptomatic outcomes as covariates.

Regarding the ICOAP Intermittent pain score, no significant associations were detected on the unadjusted analysis except for effusion at 2 months. However, the adjusted model revealed significant results at 2‐ and 12‐month follow ups (Table 5).

**TABLE 5:** Association of OMERACT ultrasound knee osteoarthritis scores with change in Intermittent and Constant Osteoarthritis Pain score Intermittent pain.

Ultrasound pathologies	Grading score	Unadjusted β (95% CI)		Adjusted β (95% CI)^a^	
		2 months	12 months	2 months	12 months
Synovitis	0–3	1.31 (−0.65, 3.28)	1.30 (−0.90 (3.50)	1.82 (0.27, 3.90)	1.68 (−0.42, 3.78)
Global synovitis	0–9	0.65 (−0.30, 1.34)	0.64 (−0.13, 1.40)	**0.91 (0.12, 1.70)**	**0.79 (0.01, 1.58)**
Synovial hypertrophy	0–1	2.58 (0.63, 5.80)	1.08 (−2.59, 4.75)	**4.46 (0.77, 8.14)**	2.23 (−1.40, 5.86)
Effusion	0–1	**3.36 (0.8, 6.64)**	1.94 (−1.84, 5.72)	**4.06 (0.31, 7.81)**	2.32 (−1.56, 6.20)
Power Doppler	0–1	1.42 (−1.89, 4.73)	1.41 (−2.28, 5.10)	**3.11 (0.89, 5.34)**	**2.60 (0.37, 4.84)**

*Note:* Bold values for significant test, *p* < .05.

^a^ Adjusted for adjusting for age, gender, body mass index, duration of disease, radiographic Kellgren–Lawrence Grade, and baseline symptomatic outcomes as covariates.

### 3.3 Ultrasound predictors for improvement in functional outcome

There was an improvement in functional outcome in the PRP cohort over 2 months (−4.2 ± 10.9) and 12 months (−5.6 ± 13.0) (Table 2). Only global synovitis showed a significant association with functional improvement at 2 and 12 months on both models (Table 6). For example, when global synovitis was present (0–9), the adjusted model revealed a functional improvement of 1.89 units (β 1.89, 95% CI 0.20–3.59) at 2 months and of 1.97 units (β 1.97, 95% CI 0.01–3.93) at 12 months on the WOMAC function sub‐score. Power Doppler exhibited a significant association at 2 months but not at 12 months on the adjusted model only.

**TABLE 6:** Association of OMERACT ultrasound knee oseoarthritis scores with change in Western Ontario and McMaster Universities Osteoarthritis function sub‐scores.

Ultrasound pathologies	Grading score	Unadjusted β (95% CI)		Adjusted β (95% CI)^a^	
		2 months	12 months	2 months	12 months
Synovitis	0–3	2.89 (−1.19, 6.97)	2.80 (−2.04, 7.64)	3.44 (−1.09, 7.98)	2.76 (−2.54, 8.05)
Global synovitis	0–9	**1.47 (0.05, 2.89)**	**1.74 (0.10, 3.38)**	**1.89 (0.20, 3.59)**	**1.97 (0.01, 3.93)**
Synovial hypertrophy	0–1	1.43 (−5.57, 8.44)	2.19 (−6.00, 10.37)	4.49 (−3.89, 12.87)	5.17 (−3.95, 14.30)
Effusion	0–1	1.22 (−5.91, 8.35)	3.99 (−4.366, 12.33)	3.21 (−5.32, 11.73)	6.45 (−3.19, 16.09)
Power Doppler	0–1	3.38 (−3.06, 9.82)	4.01 (−3.77, 11.79)	**5.88 (0.80, 10.97)**	5.72 (−0.02, 11.45)

*Note:* Bold values for significant test, *p* < .05.

^a^ Adjusted for adjusting for age, gender, body mass index, duration of disease, radiographic Kellgren–Lawrence Grade, and baseline symptomatic outcomes as covariates.

## 4 DISCUSSION

This exploratory cohort study showed that ultrasound prognostic factors representative of inflammation (especially global synovitis) were significantly associated with improvements in some pain and functional outcomes in people with mild to moderately severe knee OA undergoing a series of three weekly intra‐articular PRP injections. The associations with pain were mostly seen only in the short‐term, whereas associations with function were seen both in the short‐term and longer‐term. The presence of the association for function, but not for most pain measures, at 12 months was supported by our exploratory findings from the main RESTORE randomized controlled trial (RCT) that showed that joint effusion measured from magnetic resonance imaging did not moderate pain outcomes in a proper moderation analysis.^19^

Chen et al. demonstrated that at least two monthly PRP injections may have some benefits in patients with mild to moderate knee OA combined with ultrasound‐confirmed suprapatellar bursitis as there were significant decreases in synovial fluid volume and proteins associated with inflammation, such as apolipoprotein A‐I, haptoglobin, transferrin, and matrix metalloproteinase.^9^ PRP administration also leads to a significant reduction of matrix metalloproteinase‐13, an increase in hyaluronan synthase‐2 expression in synoviocytes, and an increase in cartilage synthetic activity compared with hyaluronic acid (HA).^11^ These findings may explain why higher inflammation before treatment as measured by ultrasound was associated with greater symptom improvements with PRP, given that the postulated mechanism of action of PRP was related to inflammation.

In the literature, we are aware of several studies that evaluated ultrasound pathologies in people receiving treatment with PRP;^32^, ^33^, ^34^, ^35^ however, these studies examined the effects of PRP on longitudinal changes of ultrasound‐detected joint features, but not whether ultrasound features measured at baseline predicted clinical outcomes as prognostic indicators, and so direct comparison with our study could not be made.

The majority of the clinical practice guidelines that included the use of PRP in knee OA are either uncertain or unable to make a formal recommendation because of the lack of high‐quality evidence^36^ and/or a current lack of standardization in PRP protocols.^22^, ^37^ A lack of clear definition and standardization of PRP products and varying costs also contribute to the uncertainty of PRP products. It is crucial that future clinical research is performed and reported with a more standardized methodology to minimize study‐to‐study variability.^38^ Future work to determine subgroups of patients who may be more responsive to PRP treatment than others is required.

Our study has several limitations. First, as our study is focused on patients with mild to moderate knee OA, the findings cannot be generalized to patients with severe knee OA. Second, the study was designed to explore the ultrasound predictors as a post‐hoc analysis within the main RCT, although the study objective was pre‐planned. Third, multiple testing was conducted, raising the possibility of a false positive. In addition, we did not adjust for paracetamol intake as rescue medication, which may influence pain reporting. Hence, a confirmatory study using a larger sample size with an RCT design could be relevant to determine whether these prognostic factors are indeed moderators of the response to intra‐articular PRP.

## 5 CONCLUSION

Ultrasound indices assessing features suggestive of knee inflammation predicted short‐term improvement in some pain outcomes and both short‐ and longer‐term improvement in a self‐reported functional outcome in people with knee OA provided with PRP injection. Whether these ultrasound indices moderate PRP treatment responsiveness needs to be evaluated.

## AUTHOR CONTRIBUTIONS

WMO, DJH, JL, and KLB contributed to study concept and design; WMO, SY, and VD acquired the data; WMO and DJH analyzed and interpreted the data. WMO drafted the first manuscript, and all the authors critically revised the manuscript for important intellectual content. WMO had full access to all of the data in the study and takes responsibility for the integrity of the data and the accuracy of the analysis.

## CONFLICT OF INTEREST STATEMENT

The authors have no conflicts of interest.

## Data Availability

Data available on request from the authors.
